# ALPS, FAS, and beyond: from inborn errors of immunity to acquired immunodeficiencies

**DOI:** 10.1007/s00277-022-04761-7

**Published:** 2022-01-20

**Authors:** Filippo Consonni, Eleonora Gambineri, Claudio Favre

**Affiliations:** 1 Anna Meyer Children’s Hospital, University of Florence, Florence, Italy; 2 Division of Pediatric Oncology/Hematology, BMT Unit, Meyer University Children’s Hospital, Viale Gaetano Pieraccini 24, 50139 Florence, Italy; 3 Department of Neurosciences, Psychology, Drug Research and Child Health (NEUROFARBA), University of Florence, Florence, Italy

**Keywords:** ALPS, FAS, Lymphoproliferation, Immune dysregulation, Primary immunodeficiencies, Double-negative T cells

## Abstract

Autoimmune lymphoproliferative syndrome (ALPS) is a primary immune regulatory disorder characterized by benign or malignant lymphoproliferation and autoimmunity. Classically, ALPS is due to mutations in *FAS* and other related genes; however, recent research revealed that other genes could be responsible for similar clinical features. Therefore, ALPS classification and diagnostic criteria have changed over time, and several ALPS-like disorders have been recently identified. Moreover, mutations in *FAS* often show an incomplete penetrance, and certain genotypes have been associated to a dominant or recessive inheritance pattern. *FAS* mutations may also be acquired or could become pathogenic when associated to variants in other genes, delineating a possible digenic type of inheritance. Intriguingly, variants in *FAS* and increased TCR αβ double-negative T cells (DNTs, a hallmark of ALPS) have been identified in multifactorial autoimmune diseases, while FAS itself could play a potential role in carcinogenesis. These findings suggest that alterations of FAS-mediated apoptosis could trespass the universe of inborn errors of immunity and that somatic mutations leading to ALPS could only be the tip of the iceberg of acquired immunodeficiencies.

## Introduction

Autoimmune lymphoproliferative syndrome (ALPS) is a rare genetic disorder of immune regulation characterized by an impairment of lymphocyte homeostasis [1]. Clinically, ALPS has been known since the 60s [2], while its first genetic characterization dates back to 1995, when the first disease-causing mutations were identified in *FAS* gene [3–5].

ALPS clinical presentation is characterized by lymphoproliferation (lymphadenopathy and/or organomegaly), autoimmune phenomena (mainly cytopenias), and an increased incidence of lymphoma [6]. Therefore, clinical management aims at monitoring patients for the development of malignancies, while non-malignant lymphoproliferation and cytopenias benefit from several types of immunosuppressants (e.g., steroids and Sirolimus) [1, 7, 8]. Immunological tests typically show increased TCR α/β CD4^-^CD8^-^ “double negative” T cells (DNTs, a hallmark of the disease) and other ALPS biomarkers, such as high levels of vitamin B12, IL-10, and sFASL and impaired FAS-mediated apoptosis [9, 10].

Clinical and laboratory features have been combined together creating diagnostic criteria for ALPS, first outlined in 1999 [11] and later revised in 2009 [9] and 2019 [12] (Tables 1, and 2). In parallel, research revealed that ALPS could also originate from germline mutations in FAS-Ligand (*FASL*) and caspase-10 (*CASP10*) genes — both involved in the extrinsic apoptosis pathway [13, 14] — and from somatic mutations in *FAS* [15]. On the other hand, a significant number of patients met the diagnostic criteria without evidence of mutations in the known causative genes. This complex genetic background brought to a classification of ALPS — first created in 1999 [11] and then revised in 2009 [9] (Table 3) — which denominated as ALPS-undetermined (ALPS-U) those cases where no known genetic defect was identified. Finally, in the last decade, alternative pathways of disease pathogenesis have been hypothesized, consistently with the identification of several genes whose mutations give rise to ALPS-like clinical phenotypes [16].

**Table 1:** Revised diagnostic criteria for ALPS (2009) [9]. *DNT* double-negative T cells.

Required	
1. Chronic (> 6 months), nonmalignant, noninfectious lymphadenopathy or splenomegaly or both	
2. Elevated CD3^+^TCRαβ^+^CD4^-^CD8^-^ DNT cells (≥ 1.5% of total lymphocytes or 2.5% of CD3^+^ lymphocytes) in the setting of normal or elevated lymphocyte counts	
Accessory	
Primary	
1. Defective lymphocyte apoptosis (in 2 separate assays)	
2. Somatic or germline pathogenic mutation in *FAS*, *FASLG*, or *CASP10*	
Secondary	
1. Elevated plasma sFASL levels (> 200 pg/mL) OR elevated plasma interleukin-10 levels (> 20 pg/mL) OR elevated serum or plasma vitamin B12 levels (> 1500 ng/L) OR elevated plasma interleukin-18 levels (> 500 pg/mL)	
2. Typical immunohistological findings as reviewed by an experienced hematopathologist	
3. Autoimmune cytopenias (hemolytic anemia, thrombocytopenia, or neutropenia) AND elevated immunoglobulin G levels (polyclonal hypergammaglobulinemia)	
4. Family history of a nonmalignant/noninfectious lymphoproliferation with or without autoimmunity	
Definitive diagnosis: presence of both required criteria plus one primary accessory criterion	
Probable diagnosis: presence of both required criteria plus one secondary accessory criterion

**Table 2:** Clinical criteria for a probable diagnosis of ALPS (2019) as defined in 2019 by the European Society for Immunodeficiencies (ESID) registry’s working definitions for clinical diagnosis of Primary immunodeficiencies (PID) [12].

At least one of the following:	
1. Splenomegaly	
2. Lymphadenopathy (> 3 nodes, > 3 months, non-infectious, non-malignant)	
3. Autoimmune cytopenia (≥ 2 lineages)	
4. History of lymphoma	
5. Affected family member	
AND at least one of the following:	
1. CD3^+^TCRαβ^+^CD4^-^CD8^-^ of CD3^+^TCRαβ^+^ T cells > 6%	
2. Elevated biomarkers (at least 2 of the following):	
• sFASL > 200 pg/ml	
• Vitamin B12 > 1500 ng/L	
• IL-10 > 20 pg/ml	
• Impaired FAS-mediated apoptosis

**Table 3:** Revised classification of ALPS (2009) [9]

Revised nomenclature	Gene	Definition
ALPS-FAS	*FAS*	Patients fulfill ALPS diagnostic criteria and have germline homozygous or heterozygous mutations in *FAS*
ALPS-sFAS	*FAS*	Patients fulfill ALPS diagnostic criteria and have somatic mutations in *FAS*
ALPS-FASL	*FASL*	Patients fulfill ALPS diagnostic criteria and have germline mutations in *FASL* (FAS ligand)
ALPS-CASP10	*CASP10*	Patients fulfill ALPS diagnostic criteria and have germline mutations in *CASP10* (Caspase 10)
ALPS-U	Unknown	Patients meet ALPS diagnostic criteria; however, genetic defect is undetermined (no FAS, FASL, or CASP10 defect)

In addition to its genetic characterization, modalities of inheritance of ALPS are controversial. Both autosomal-dominant and autosomal-recessive types of inheritance have been described [17], while increasing reports of somatic mutations in *FAS* gene — detected in TCR αβ DNT cells — jeopardized the idea of ALPS as an exclusively inborn error of immunity [18]. Hence, a double-hit mechanism (i.e., predisposing germline mutations followed by disease-triggering somatic mutations) has been hypothesized [19]. Moreover, the variable penetrance seen in ALPS patients’ pedigrees [20] suggested that a digenic model of inheritance could be applicable, due to a possible role of disease-modifying genes [21].

Finally, variants in *FAS* and *FASL* have been reported in patients displaying multifactorial autoimmune diseases and cancer (e.g., systemic lupus erythematosus, SLE) [13, 22–24]. Such findings could indicate that genetic defects of lymphocyte apoptosis may be a possible underlying mechanism of autoimmunity and carcinogenesis [17, 22]. The crossroad between immune dysregulation and multifactorial autoimmunity is furtherly highlighted by reports of increased TCR αβ DNTs in patients with SLE and other autoimmune disorders [25], as well as in rare genetic disorders of immune regulation, such as STAT3 gain of function (STAT3-GoF), CTLA-4 haploinsufficiency (CHAI), and others [16, 26–29].

Herein, we review ALPS genetic background and possible relationships between disease-associated mutations and its type of inheritance. We will also describe current knowledge about somatic mutations in *FAS*, together with their possible underestimation and a potential disease-contributing role in multifactorial autoimmune disorders. Finally, we will outline current treatment options for ALPS and ALPS-related diseases.

## ALPS: one disease, many genes

### ALPS classification

**Fig. 1:**
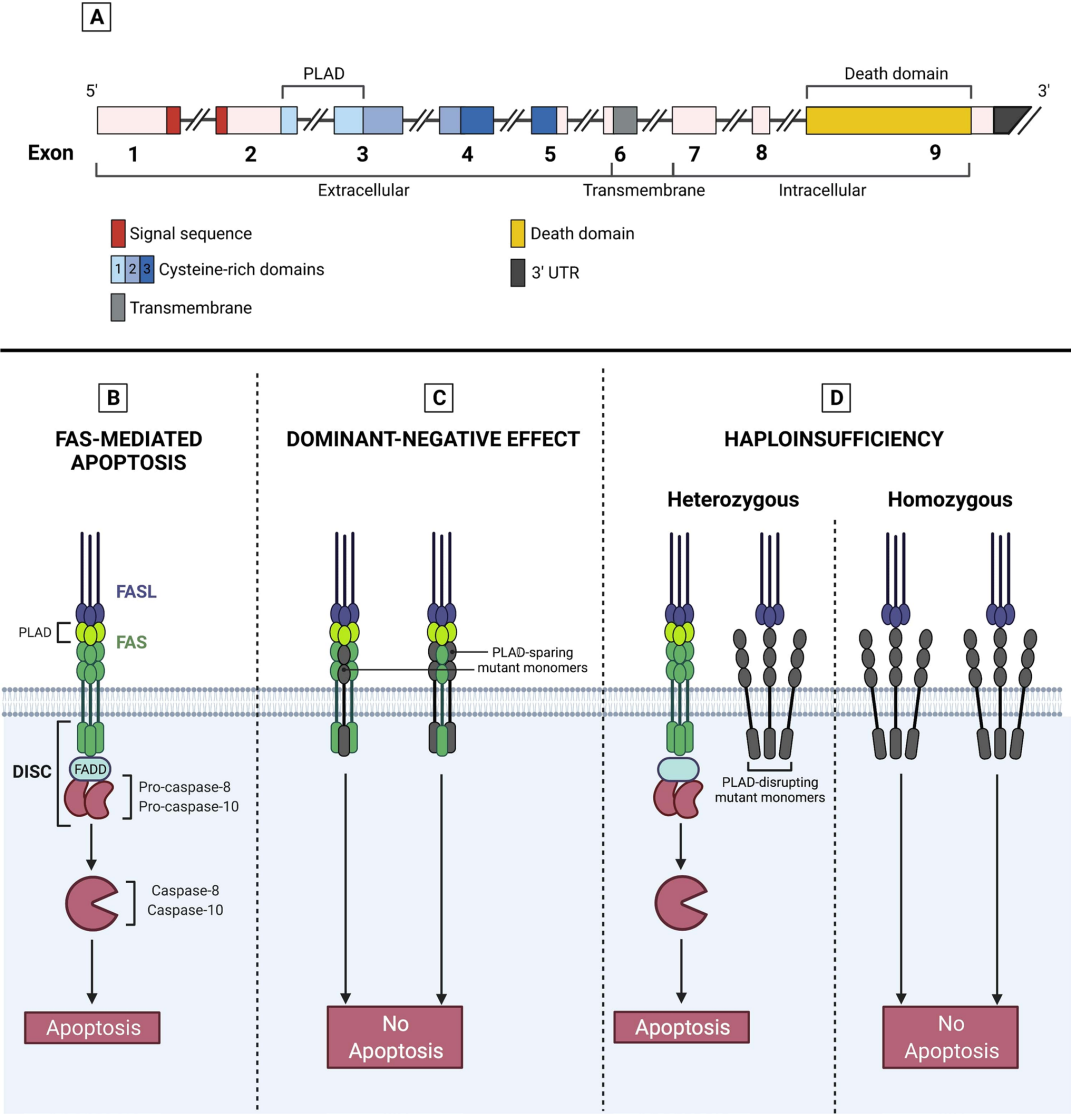
FAS structure, biology, and molecular mechanism of *FAS* mutations. **A** Intron-exon structure of *FAS* gene delineating the exons coding for the extracellular, transmembrane, and intracellular portions of FAS. Cysteine-rich domain 1 (CRD1) is also known as pre-ligand assembly domain (PLAD) and allows homotrimerization of FAS monomers. UTR, untranslated region. **B** Biological mechanism of extrinsic, FAS-mediated, pathway of apoptosis. FAS is a homotrimeric receptor expressed at the surface of many cell types, whose correct trimerization is mediated by PLAD. Binding of cognate ligand FASL allows the recruitment of FAS-associated death domain (FADD), an adaptor protein that bridges the death domain of FAS to pro-caspases-8 and caspase-10, leading to the generation of death-inducing stimulating complex (DISC). DISC formation allows the downstream activation of the caspases, resulting in a biochemical cascade that ultimately leads to apoptosis. **C** *FAS* mutations exerting a dominant-negative effect do not impair the expression of mutated, PLAD-sparing, monomers (in gray) at the cell surface. Such monomers may interact with wild-type ones in a 1:2 or 2:1 ratio, resulting in non-functional trimers that impair downstream activation of apoptosis. These mutations are clinically penetrant and usually all affected individuals develop ALPS. **D** *FAS* haploinsufficient mutations usually involve PLAD. These mutations completely impair FAS expression at the cell surface only when both alleles are affected (either because of germline homozygosity or somatic events on the second allele). In heterozygous individuals, the wild-type allele preserves FAS expression and its correct functionality, leading to apoptosis

Genes included in ALPS classification (Table 3) retrace those implicated in the extrinsic, FAS-mediated, pathway of apoptosis [9]. Briefly, FAS (also known as CD95 or tumor necrosis factor receptor superfamily member 6 — TNFRSF6) is a homotrimeric receptor whose binding to FASL homotrimers activates the intracellular death domains of FAS molecules. These domains recruit the adaptor protein FADD (FAS-associated death domain) and ultimately bind caspase-8 and caspase-10 (CASP8-10), leading to the generation of a death-inducing signaling complex (DISC). Finally, DISC activates downstream effector caspases, paving the way for the occurrence of programmed cell death [30] (Fig. 1B).

Germline mutations in *FASL* and *CASP10*, together with germline or somatic mutations in *FAS*, give rise to typical ALPS clinical and laboratory features [18, 20, 31, 32] and have therefore been included in ALPS classification [9]. On the other hand, mutations in *FADD* and *CASP8* lead both to an impairment in FAS-mediated apoptosis and a profound susceptibility to infections [33, 34]. Indeed, these genes underlie other immunological pathways leading to pleiotropic effects that trespass ALPS phenotype. Therefore, mutations in *CASP8* — formerly labeled as ALPS IIb [34] — have been removed from current ALPS classification and termed caspase eight deficiency state (CEDS), while FADD deficiency has never been actually defined as ALPS.

Regardless of the genes involved, specific criteria need to be satisfied in order to achieve a diagnosis of ALPS. These have been recently updated by the European Society for Immunodeficiencies (ESID) (Table 2) [12]. If compared to the previous ones [9], increased TCR αβ DNTs are not anymore mandatory, while the TCR αβ DNT ratio threshold has been raised from 2.5 to 6% of total TCR αβ CD3+ T cells. Moreover, immunohistological findings have been excluded, and the identification of pathogenic mutations is not needed to define ALPS, though of course necessary in order to attain a molecular diagnosis [12]. Growing relevance has been given to biomarkers, that — once combined — demonstrated to bear elevated positive and negative predictive values for ALPS [35, 36]. In particular, the combination of normal TCR αβ DNTs and in vitro apoptosis assay can essentially rule out ALPS [36, 37].

### ALPS-U: determining the undetermined

Since its first definition, 20–30% of ALPS cases lack a molecular diagnosis (ALPS-U) [9, 37]. Recent studies showed that patients with ALPS-U display a different biomarker profile, compared to those affected by ALPS-FAS [35, 36]. In detail, Molnar et al. demonstrated that ALPS-FAS is characterized by significantly higher proportions of TCR αβ DNTs, in vitro apoptosis alterations, and soluble FAS-Ligand (sFASL) levels [36]. The biological background of ALPS-U might therefore be different from classical ALPS. In accordance with this, next-generation sequencing analyses in these patients identified pathogenic or likely pathogenic mutations in signal transducer and activator of transcription 3 (*STAT3*), inhibitor of nuclear factor kappa B kinase regulatory subunit gamma/NF-kappa-B essential modulator (*IKBKG/NEMO*), perforin 1 (*PRF1*), and recombination activating 1 (*RAG1*). Such findings are consistent with ALPS-like features previously described in patients carrying defects in the same genes, even though the overall clinical phenotypes of these disorders are extremely heterogeneous and different from ALPS [38–44].

Nevertheless, in the vast majority of ALPS-U cases, an underlying genetic defect is unknown. Many genes have been recalled as hypothetic players in ALPS-U pathogenesis [16]. In order to be called ALPS-U, however, the patient’s phenotype must respond to the above-mentioned diagnostic criteria. Therefore, together with clinical features, either increased TCR αβ DNTs or two biomarkers (including impaired FAS-mediated apoptosis) must be displayed. In line with this, several candidates standing behind an ALPS-U phenotype have been described in the past decades. First of all, multiple cases of lymphoproliferation, autoimmunity, and apoptosis defect but normal TCR αβ DNTs without a genetic explanation have formerly been described as Dianzani autoimmune lymphoproliferative disease (DALD) [45, 46]. Later, germline gain-of-function (GoF) mutations in NRAS or somatic GoF variants in KRAS were found, depicting a phenotype called RAS-associated autoimmune lymphoproliferative disease (RALD), characterized by intrinsic apoptosis defect and normal or slightly elevated TCR αβ DNTs [47, 48]. Increased TCR αβ DNTs and autoimmune features were also found in STAT3-GoF, CHAI and X-linked immunodeficiency with magnesium defect, Epstein-Barr virus (EBV) infection, and neoplasia (XMEN), demonstrating that ALPS-U may comprise a wide variety of responsible genes [26, 28, 29].

Finally, we can speculate that somatic mutations in *FAS* [18] or in other related genes could be responsible for a significant portion of ALPS-U cases. These could potentially be underestimated, given that somatic mutation analysis is not easily feasible, since patients’ samples are often not enough to perform TCR αβ DNTs sorting [35]. Future developments in this field will hopefully clarify the impact of somatic mutations on ALPS-U pathogenesis.

## ALPS-FAS: one gene, many inheritance patterns

*FAS* was the first gene associated with ALPS [3, 4]. Since its identification, hundreds of mutations in it have been described, accounting for roughly 70% of ALPS cases [49]. *FAS* gene consists of an extracellular portion (exons 1–5), a transmembrane domain (exon 6), and an intracellular part (exons 7–9) [50]. The extracellular portion contains 3 cysteine-rich domains (CRDs): while CRDs 2–3 are paramount to bind FASL, CRD1 allows homotrimerization of FAS molecules and is therefore named pre-ligand assembly domain (PLAD). On the intracellular side, exon 9 encodes for the death domain (DD), which is critical for FADD binding and downstream activation of apoptosis [8] (Fig. 1A).

In the last two decades, mutations have been described throughout any site of FAS: the majority (70%) of ALPS-FAS cases are due intracellular FAS mutations, while 50% involve the DD [8]. Increasing reports speculated the existence of a relationship between genotype and inheritance pattern. This implies that different mutations exert distinct molecular effects, leading to either dominant or recessive types of inheritance [17]. Moreover, the discovery of somatic *FAS* mutations in TCR αβ DNTs furtherly complicated this model [15]. Below, we attempt to clarify these important aspects.

### Dominant inheritance in ALPS-FAS

Studies on mice strains *Ipr* [51] and *Ipr*^cg^ [52] initially allowed to understand the role of *Fas* gene, the murine analog of *FAS* [21]. *Ipr*^cg^ strains beared mutations in the DD of Fas: importantly, their inheritance pattern proved to be not fully recessive as in *Ipr* mice [52]. Similarly, a dominant-interfering effect has been described since the first human reports of ALPS-FAS [4]. Later, mutations in the intracellular portion of FAS that did not abolish its surface expression were considered to be responsible for a dominant type of inheritance [53]. Finally, Siegel et al. shed more light on this aspect, establishing that PLAD preservation was always correlated with the presence of a dominant-negative mechanism [54].

According to this model, transmembrane wild-type FAS combines with the mutated form (via PLAD-PLAD interactions) in a 2:1 or 1:2 ratio. This results in a trimer which is unable to recruit FADD after binding of cognate ligand FASL. Hence, the majority of FAS trimers display defective intracellular signaling, even though 50% of total FAS in the cell is in a wild-type form [55] (Fig. 1C).

Recently, a large retrospective study demonstrated that a dominant-negative effect stands behind the majority of ALPS-FAS cases [8], justifying a dominant type of inheritance (i.e., one mutation leads to ALPS) [17]. Moreover, reports of single-allele somatic dominant mutations in FAS confirmed that this important molecular mechanism is also conserved in ALPS-sFAS [18, 56].

### Recessive inheritance in ALPS-FAS

In contrast to *Ipr*^cg^ mice, murine *Ipr* strains bore mutations in intron 3, leading to reduced Fas expression and behaving as a recessive defect [21, 51]. Later on, several examples of autosomal recessive ALPS-FAS have been reported, though less frequently than cases with a dominant type of inheritance [53, 57, 58].

These patients displayed either mutations in the extracellular portion of FAS (which particularly involved PLAD) [8, 54] or in the transmembrane domain (i.e., exon 6) [55]. In any case, surface expression of FAS was abolished, due to impaired trimerization. This implies that mutated monomers cannot anymore bind their wild-type (wt) homologue, which may be undisturbedly displayed on the cell surface. This situation jeopardizes a dominant-interfering effect, while the presence of two mutated alleles gives rise to haploinsufficiency [59, 60] (Fig. 1D). In line with this, FAS-wt transfection in FAS-mutated cell cultures both corrected the apoptosis defect and increased surface FAS expression, demonstrating the hypothesis of a haploinsufficiency mechanism [59].

Nevertheless, germline haploinsufficient mutations were found both in patients and asymptomatic relatives, and this fact did not find an immediate explanation. Initially, a reduced penetrance due to this genotype was hypothesized [61]. The conundrum was partially solved by the identification of somatic events in patients, while not in asymptomatic carriers, with familial haploinsufficient mutations [19]. Therefore, these defects are non-penetrant, and an additional event on the second allele is needed in order to determine clinical manifestations [17]. Such “second hit” may either be inherited [53, 57] or — more frequently — acquired [19, 62, 63], as furtherly described.

### Somatic mutations in FAS (ALPS-sFAS)

Somatic heterozygous dominant mutations in *FAS* were initially reported in 2004 [15], depicting for the first time the role of somatic mutations in a non-malignant disease [17]. Such variants were recognized in patients’ TCR αβ DNTs, which were found to be increased, just as in germline ALPS-FAS. Clinical features were also not distinguishable but — compared to germline ALPS-FAS — patients with somatic mutations did not display a severe impairment in FAS-mediated apoptosis [18]. Such finding has been repeatedly confirmed [1] and bears a possible explanation in the fact that TCR αβ DNTs do not survive in cell culture, with only a fraction of mononuclear cells being actually mutated. This fraction is apparently sufficient to give rise to clinical features, but not enough to significantly impair apoptosis [18].

Further studies revealed that somatic *FAS* mutations are not rare and represent up to 15% of total ALPS cases [63]. Indeed, these variants respond to the same molecular mechanisms described above. Therefore, dominant-interfering somatic mutations alone are sufficient to induce the disease [15, 56], while patients displaying germline haploinsufficient mutations require a somatic event in order to develop ALPS clinical features [19, 56]. The latter, moreover, exhibit symptoms at a later stage [56], coherently with the fact that somatic mutations initially occur in hematopoietic stem cells (HSCs) and furtherly provide a selective advantage to mutated cells. These lymphocytes, however, require several years of proliferation in order to reach sufficient numbers to have a clinical impact [17].

We may speculate that somatic mutations in *FAS* are probably underdiagnosed, for a couple of reasons. First, TCR αβ DNTs sorting is often challenging to perform and such difficulty discourages single-cell DNA sequencing [21, 35]. Moreover, somatic mutations are potentially responsible for disease penetrance in many patients displaying heterozygous haploinsufficient germline *FAS* mutations. However, TCR αβ DNTs sorting and sequencing is seldom performed in these cases, since clinicians have already achieved a genetic diagnosis. Finally, somatic variants may potentially lie at the base of more common, multifactorial, autoimmune disorders [17].

## Double-hit hypothesis in ALPS: one disease, two mutations needed

**Fig. 2:**
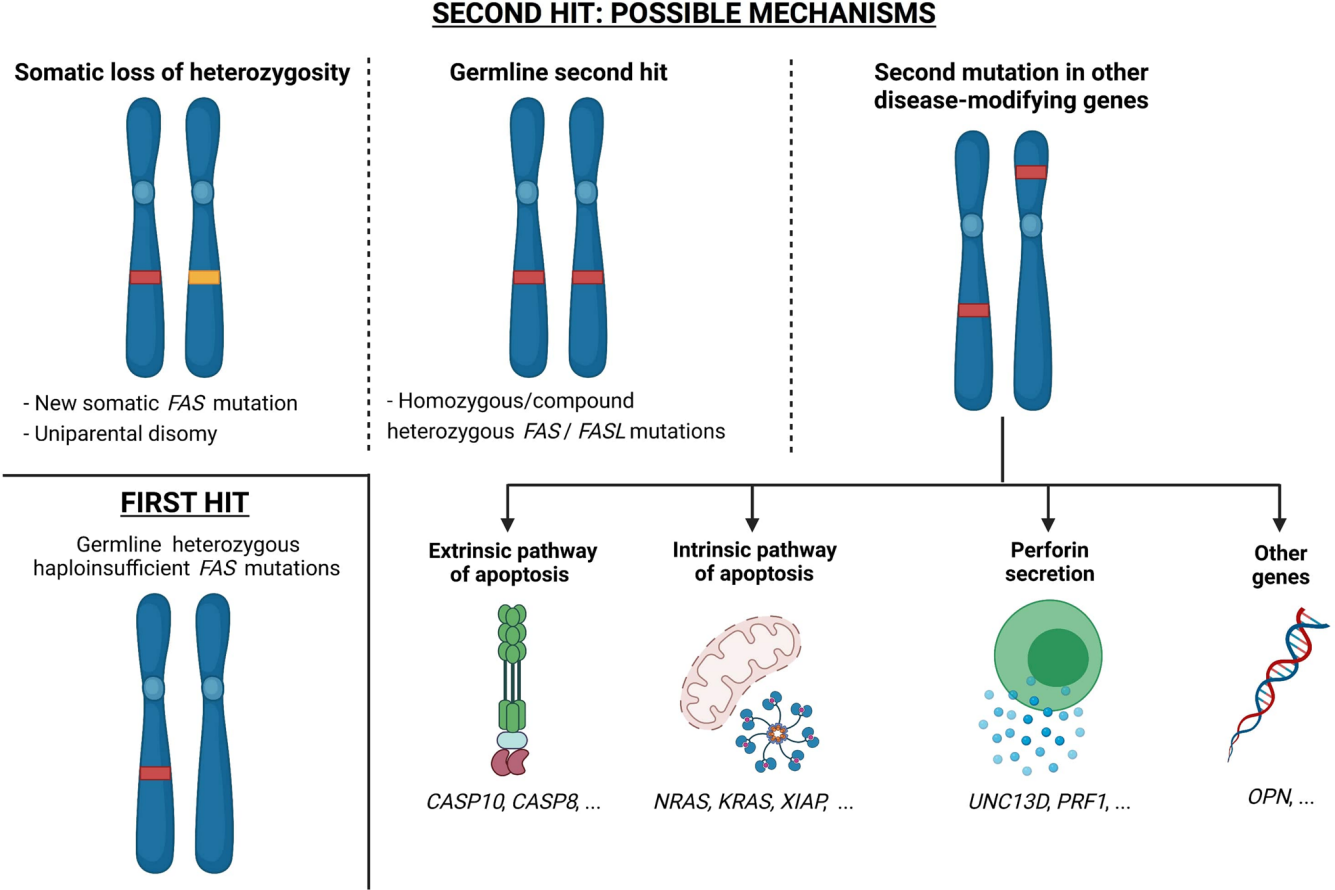
Double-hit hypothesis in ALPS. Germline heterozygous haploinsufficient mutations in FAS are not sufficient to determine the disease: a second hit is needed in order to develop ALPS. Different mechanisms may account for the second hit. First, a somatic event in the second *FAS* allele could be possible (e.g., a new acquired mutation or uniparental disomy). Second, germline homozygous or compound heterozygous mutations in *FAS* or *FASL* can determine ALPS. Finally, a second mutation in other disease-modifying genes could induce the disease, depicting a digenic inheritance pattern. These genes may involve both the extrinsic and intrinsic pathway of apoptosis [47, 48, 71, 73, 74, 77], perforin secretion [38, 76], or other possible mechanisms [78, 79]

ALPS patients’ pedigrees often show an incomplete disease penetrance [20, 63]. However, additional genetic mechanisms may come into play and ultimately determine the expression of an ALPS disease phenotype [1, 17]. These predisposing mutations act as a “second hit” and include somatic *FAS* mutations (leading to loss of heterozygosity, LoH), a second germline mutation (leading to homozygous/compound heterozygous genotypes), or variants in other disease-modifying genes (Fig. 2).

### Somatic loss of heterozygosity in FAS

ALPS has been recently summoned as a paradigm of inborn errors of immunity due to a somatic mosaicism [64, 65]. Indeed, evidence suggests that ALPS may respect a “two-hit” mechanism similar to the one postulated in the 70s for retinoblastoma’s pathogenesis [66]. In line with this, ALPS was described in 7 patients bearing germline mutations in the extracellular domain (ECD) of *FAS*, together with a somatic event detected in the second *FAS* allele in TCR αβ DNTs [19]. Such somatic event is not necessarily a new mutation, but often corresponds to uniparental disomy that makes the cell homozygous for the original germline variant [17, 56].

Not surprisingly, ALPS-causing somatic events are almost exclusively identified in individuals bearing haploinsufficient mutations, which ideally behave in a recessive fashion [63]. Among these subjects, only those displaying a “second hit” actually develop an ALPS phenotype [62], which typically manifests at an older age [56]. However, not every patient with ALPS and a haploinsufficient mutation was found to bear a somatic event. This could be potentially due to intronic *FAS* mutations or variants in other disease-modifying genes [56].

### Germline double-hit

In rare instances, both hits on the *FAS* gene may be inherited. This often leads to severe phenotypes and may be due either to compound heterozygous or homozygous *FAS* mutations (formerly described as ALPS type 0) [1, 57, 58, 67]. The effect of the mutation (i.e., haploinsufficiency or dominant-negative) is trivial in these cases, since both alleles are involved.

Similarly, ALPS-FASL supposedly observes an autosomal recessive inheritance, giving rise to severe clinical phenotypes [31, 32, 68, 69]. However, ALPS-FASL inheritance may also behave outside the box, since a dominant-negative effect has been reported [70] and heterozygous mutations have been associated to SLE [13]. The precise mechanism hiding behind FASL mutations is still not clear, given that these reports are extremely rare [17].

### Second hit in disease-modifying genes

Studies in mice originally noted that double heterozygous subjects (i.e., *lpr/+, gld/+*) for murine analogs of *FAS* and *FASL* could develop lymphoproliferation and autoimmunity [52]. In the last two decades, reports of ALPS and ALPS-like patients revealed that *CASP10* and other genes may influence disease pathogenesis. These observations implicated that ALPS could be inherited in a digenic or oligogenic fashion, thus trespassing a classical Mendelian transmission model [17, 71].

#### CASP10

Only two pathogenic mutations in *CASP10* have been identified thus far (i.e., p.I406L and p.L258F), both leading to a dominant-negative effect [14, 72, 73]. Conversely, several other variants or polymorphisms in *CASP10*, whose pathogenicity is still controversial, have been described [14, 73, 74]. Nevertheless, multiple reports of *FAS*-mutated individuals bearing a concomitant *CASP10* variant and developing ALPS clinical features suggested the existence of an underlying digenic mechanism [71, 74, 75]. Both FAS and CASP10 belong to the same — extrinsic — apoptosis pathway; therefore, mutations in these genes would produce complementary effects, leading to impairment of programmed cell death. Similarly, some *CASP10* variants may also display a protective effect towards the development of ALPS [72]. Hence, interactions between variants in genes belonging to the same pathway may be both synergic and antagonistic. Finally, ALPS or ALPS-like features may also arise from the complementary effect of variants in *CASP10* and in another non-*FAS* gene, such as *CASP8* or *TNFRSF13C* (i.e., BAFF receptor, whose mutations are typically associated with common variable immunodeficiency, CVID) [73].

#### Other disease-modifying genes

In the last two decades, interesting reports revealed that the “second hit” in ALPS pathogenesis may concern other genes, beyond those involved in the extrinsic apoptosis pathway. For instance, variants in *UNC13D* and *PRF1* highlighted that genes involved in perforin secretion by natural killer (NK) and cytotoxic T lymphocytes (CTLs) may bestow clinical significance to certain heterozygous *FAS* mutations [38, 76]. A similar role could be played by genes belonging to the intrinsic apoptosis pathway, such as *XIAP* [77] or *NRAS* and *KRAS*. Mutations in these last two genes may alone give rise to RALD [47, 48], but we may speculate that variants in them could also determine ALPS in genetically predisposed individuals. Moreover, since specific osteopontin haplotypes have been formerly related to DALD [78], a possible role of *OPN* gene in ALPS could be contemplated. Finally, other biological mechanisms, such as microRNA overexpression (e.g., miR-146a), are involved in Fas downregulation in mice and could ideally be involved in ALPS pathogenesis [79].

## ALPS, cancer, and autoimmunity: one mechanism, many diseases

**Fig. 3:**
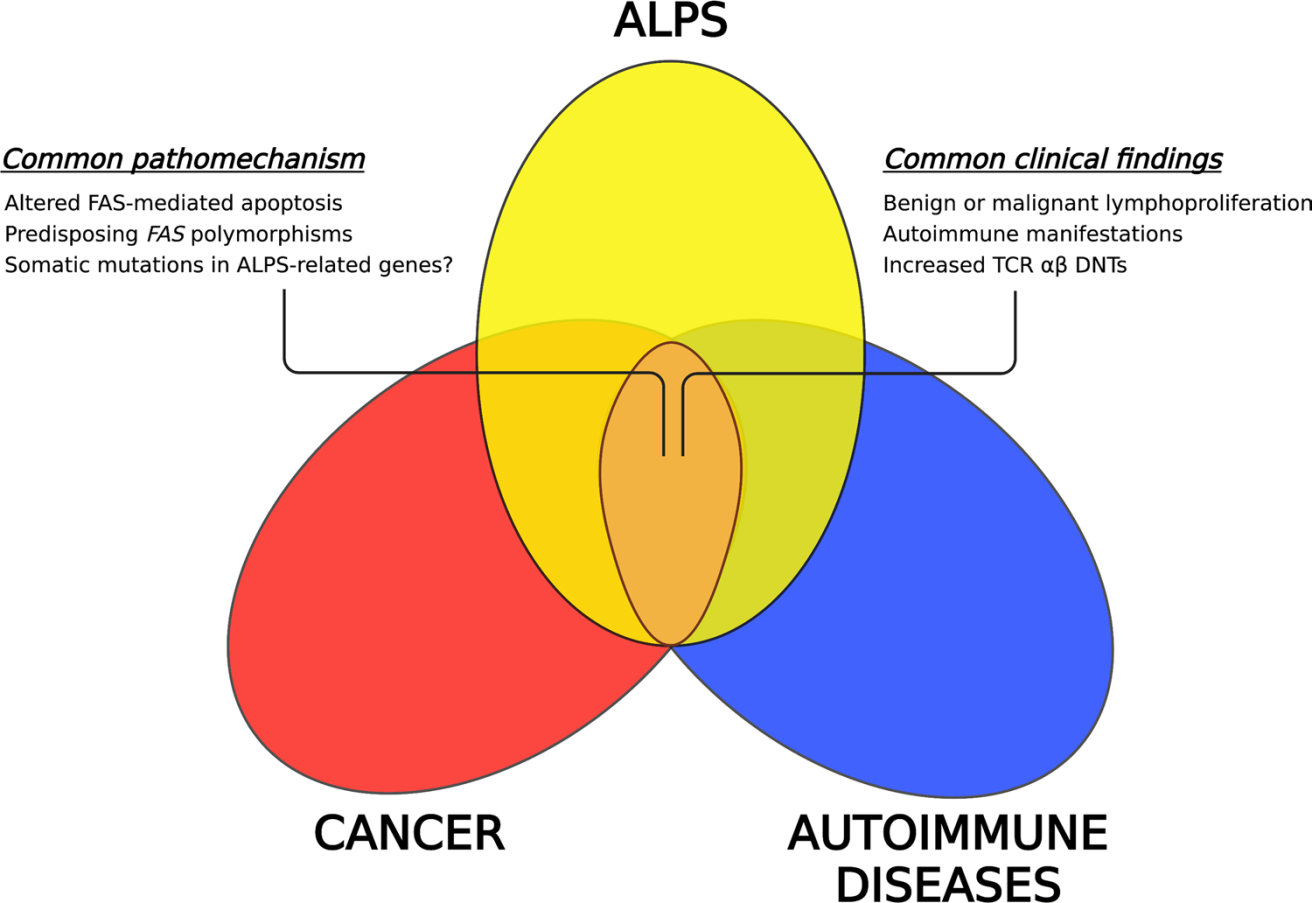
ALPS, cancer and autoimmune diseases. Venn diagram showing mutual relationships and shared clinical and pathophysiologic features connecting ALPS to more common conditions such as cancer and autoimmune diseases. TCR αβ DNTs, T-cell receptor αβ double-negative T cells

Alterations in the extrinsic pathway of apoptosis are not prerogative of ALPS but may also hide behind more common conditions such as cancer and autoimmune diseases [21, 22]. Indeed, research in the last 10 years revealed that single-nucleotide polymorphisms (SNPs) in *FAS* or *FASL* may correlate with some types of cancer and multifactorial autoimmune diseases [23, 80, 81]. Similarly, an expansion of TCR αβ DNTs is not exclusive of ALPS but may also be found in other conditions [25], where these cells may play pathogenic roles [82]. Therefore, the genetic and immunological features of ALPS push beyond the limits of this disease and involve more common clinical situations (Fig. 3). In this section, we try to summarize this cutting-edge topic.

### FAS and cancer

While initially only non-malignant lymphoproliferation was taken into account in ALPS definitions [9, 11], the latest diagnostic criteria comprise a history of lymphoma as a major clinical feature (Table 2) [12]. In parallel, recent research highlighted potential carcinogenic implications of FAS, whose role is still controversial [22].

Most cancer cells are resistant to FAS-mediated apoptosis [22]. Such an escape mechanism is required since the CD95/CD95L interaction may destroy cells by inducing DISC formation [83]. However, baseline FAS signaling seems crucial for cancer cell survival [84], since a complete elimination of CD95/CD95L interplay leads to an irreversible cell death called DICE (death induced by CD95R/L elimination) [22]. Such peculiar behavior might be explained by non-apoptotic functions of FAS, whose engagement also promotes inflammation and carcinogenesis [83]. In light of this, preclinical studies on therapies targeting the FAS/FASL pathway have been implemented [85], even though severe hepatotoxicity hampers their clinical applicability [22].

In a clinical setting, lymphoma may be a threatening part of the natural history of ALPS. Interestingly, this malignancy may also develop in healthy *FAS*-mutated relatives of ALPS patients [8]. In a wider perspective, a relationship between cancer risk and *FAS* polymorphisms is controversial [23, 86–88]. A meta-analysis argues for a reduction of cancer risk in individuals bearing FAS-1377 G/A SNP [86]. Similarly, a previous hypothetical association between FAS-670 A/G SNP and acute myeloid leukemia (AML) has been rejected [88]. On the other hand, SNPs in codons FAS-1377 and FASL-844 correlated with bladder cancer [81] and neuroblastoma [23]. Further research may shed more light on these aspects and allow to identify possible factors determining FAS-mediated carcinogenesis.

### FAS, FASL, and autoimmunity

B cell differentiation is typically altered in ALPS-FAS [1]. Murine models showed that an impaired germinal center reaction inappropriately allows survival of autoreactive B cells [89]. Therefore, along with defective T cell apoptosis, a dysregulated B cell compartment is characteristic of ALPS.

Such immunological background is coherent with a clinical overlap among ALPS, CVID [90] and — most importantly — SLE [91, 92]. In particular, lupus and lupus-like features may characterize the clinical course of ALPS-FAS and ALPS-FASL [13, 92]. Moreover, a role for apoptosis in SLE susceptibility is plausible, since SNPs FAS-670 A/A and FASL-844 C/C (alone or combined in a digenic type of inheritance) are associated with an increased risk of lupus [24, 93]. On the other hand, variants in other genes involved in apoptosis (i.e., BAX) seem to be protective towards the development of SLE [93]. Other autoimmune diseases have been associated with SNPs in *FAS* and these include Hashimoto’s thyroiditis, systemic sclerosis, and multiple sclerosis [80, 94].

Apart from polymorphisms, the increasing discovery of somatic mutations in *FAS* opens an intriguing debate. Somatic variants in this and other genes could potentially account for the clinical discordance of monozygotic twins, a frequent phenomenon in autoimmunity whose explanation has been classically attributed to epigenetic modifications [95]. Future research could reveal if *FAS* somatic mosaicism hides behind those autoimmune conditions that interestingly display an ALPS-like expansion of TCR αβ DNTs [25].

### DNTs in ALPS and autoimmune diseases

TCR αβ DNTs are a peculiar T cell subset whose origin and pathogenetic role has not been clearly elucidated [82, 96]. Initially, TCR αβ DNTs were thought to have a thymic origin [97]. However, the expression of both the senescent marker CD57 and CD45RA makes them more similar to terminally differentiated effector T cells re-expressing CD45RA (TEMRA) [98]. Such phenotype inclines towards the hypothesis that TCR αβ DNTs may peripherally derive from downregulation of CD8 in autoreactive CD8+ T cells [96, 99]. Their pathogenic role is controversial since TCR αβ DNTs are not only a hallmark of ALPS [100, 101], but have also been described in pro-inflammatory contexts (e.g., in SLE and other autoimmune diseases) or as mediators of immune regulation (e.g., in graft-versus-host disease — GVHD) [82, 102].

In ALPS, TCR αβ DNTs are not only a key feature for diagnostic purposes, but they also seem to play a relevant role in disease pathogenesis [100, 101]. An expansion of TCR αβ DNTs is tightly associated with disease development [82, 103], and their number often correlates with the presence of autoantibodies [87, 104]. Recently, an elegant study by Maccari et al. shed more light on this topic, revealing two main populations among TCR αβ DNTs: FAS-controlled DNTs (FC-DNTs, CD38+ CD45RA+) and conventional DNTs (cDNTs, CD45RA+/− CD38-) [101]. The former are IL-10 producers and are the real hallmark of ALPS-FAS, where they represent a significant proportion of total TCR αβ DNTs (usually > 25%). Moreover, these FC-DNTs share a transcriptional profile with certain CD28 + CD57+ single-positive T cells (SPTs, both CD4+ or CD8+), corroborating the hypothesis that TCR αβ DNTs derive from downregulation of CD4/CD8 coreceptor at a late differentiation step in T cell development. On the other hand, cDNTs are closely related to “canonical” CD8+ T cells, since they express both interferon-γ (IFNγ) and cytolytic molecules (granzyme B and perforin), oppositely to FC-DNTs. Unexpectedly, both TCR αβ DNT populations have been found also in healthy subjects, indicating that these cell types may also play a physiologic role. Moreover, treatment with Rapamycin led to a decrease in both ALPS and healthy subjects-derived FC-DNTs, highlighting that the maintenance of this lymphocyte subset tightly depends on mammalian target of rapamycin (mTOR) signaling [101].

Such recent findings have not yet been applicated to more common, multifactorial autoimmune diseases (e.g., SLE). Anyhow, in these disorders, TCR αβ DNTs have been identified as main actors of an increased production of the pro-inflammatory interleukin-17A (IL-17A). Such process seems to be mediated by the transcription factor cAMP-responsive element modulator (CREM) α [96]. CREMα also plays a significant role in the downregulation of CD8 from autoreactive CD8+ T cells [82, 96], which may take place in an inflammatory milieu, such as the one seen in spleens of SLE patients due to the lack of the tolerogenic splenic marginal zone macrophages (MZMs) [105]. In addition, MZMs express scavenger receptors that efficiently clear apoptotic and necrotic cellular fragments, avoiding the generation of autoimmunity against these debris [106]. Consistently, a lack of MZMs and an increase in TCR αβ DNTs are particularly displayed in SLE, where TCR αβ DNTs showed to promote the production of anti-dsDNA antibodies [96]. In a clinical context, association studies and pathologic specimens also revealed a possible role of TCR αβ DNTs in Sjögren’s syndrome [107], psoriasis [108], axial spondylarthritis [109], mixed connective tissue disease, juvenile idiopathic arthritis, juvenile dermatomyositis [110], and Behçet’s disease [111].

In contrast to the findings above, evidence suggests that at least a subset of TCR αβ DNTs may exhibit a regulatory activity [82, 112]. Even though specific markers allowing their identification are lacking, these TCR αβ DNTs have been called double-negative regulatory T cells (TCR αβ DN Tregs). Despite what the name suggests, DN Tregs are FOXP3- and must not be confused with CD4+ CD25+ FOXP3+ regulatory T cells [113]. A first interesting role of TCR αβ DN T regs was demonstrated in non-obese diabetic (NOD) mouse models, where this cell type lowered the risk of developing islet autoimmunity through the production of IL-10 [114]. In addition, TCR αβ DN T regs seem to play a significant role in the development of immune tolerance after hematopoietic stem cell transplantation (HSCT). Initially, murine models revealed that TCR αβ DN T regs were able to inhibit natural killer (NK) cell-mediated rejection of allogenic bone marrow in a perforin-dependent manner [115]. Moreover, clinical studies showed an inverse correlation between TCR αβ DNTs frequency and the risk of developing GVHD [116, 117]. Proposed mechanisms of TCR αβ DN T regs functioning are IL-10 production, the peculiar use of IFNγ to regulate immune responses, and the acquisition of alloantigens from dendritic cells (i.e., trogocytosis) in order to present them to antigen-specific CD8+ T cells and killing them through Fas-dependent apoptosis [113]. However, such immunological processes still need to be fully clarified by further studies [82].

## ALPS and ALPS-like disorders: one phenotype, many treatments

### Treatment options and management of ALPS

The most frequent presentation of ALPS is benign lymphoproliferation; however, despite potentially massive manifestations during childhood, lymphadenopathies and splenomegaly generally shrink spontaneously with age [21]. On the other hand, malignant lymphoproliferation may develop at any time during follow-up [118, 119]. Nonetheless, autoimmune cytopenias occur in more than 80% of ALPS-FAS patients and often represent a therapeutic challenge [8, 21]. Therefore, management of ALPS should cover all these aspects, and clinicians should choose treatment options taking into account that they might be administered for a lifelong span [7].

Treatment of isolated benign lymphoproliferation for cosmetic reasons is not usually indicated [7]. In case of symptoms or concomitant cytopenias, the only drug that demonstrated to significantly diminish lymphoproliferation is Sirolimus [120–122], consistently with the description of a hyperactive mTOR pathway in ALPS [123]. Splenomegaly should be carefully managed using thermoplastic spleen guards, in order to avoid splenic rupture, allowing children to participate to sport programs [7]. ALPS is characterized by poor anti-polysaccharide response and disorganized splenic marginal zone [124], correlated with a high risk of streptococcal sepsis [8]. Therefore, in order to preserve a minimal anti-polysaccharide response, splenectomy is contraindicated and should be taken into consideration if it is the only remaining therapeutic option. However, it is possible to deal with already splenectomized ALPS cases. Asplenic ALPS patients should receive long-term antibiotic prophylaxis (e.g., penicillin V) and periodic anti-pneumococcal reimmunization every 4–5 years [7].

Autoimmune cytopenias in ALPS have been classically treated as sporadic immune cytopenias, using corticosteroids and intravenous immunoglobulins (IVIG) as first-line options [7, 125–127]. Due to refractoriness to these treatments, cytopenias in ALPS often require the use of second-line agents. Rituximab and/or Mycophenolate mofetil (MMF) were the first studied, the former being particularly effective for thrombocytopenia, though not usually recommended due to a consistent increase in the risk of severe infections [128]. On the other hand, MMF proved to be an effective steroid-sparing agent, without evidence of significant toxicities or infections [7, 129]. However, further evidence revealed a dramatic effectiveness of Sirolimus [120, 121], which may be considered a targeted treatment for ALPS [123], consistently with the high degree of remission achieved and the significant reduction of ALPS biomarkers after 6 months of therapy [122]. For these reasons, Sirolimus may be considered as a first-line treatment option, and, once complete remission is achieved, its serum levels may be maintained at a lower therapeutic range (i.e., 2–5 ng/mL) [122].

ALPS patients exhibit an increased risk of lymphoma; therefore, periodic surveillance with CT and positron emission tomography (PET) scans should be carried out, and lymph node biopsies must be performed in case of clinical or radiological suspect of malignancy [7]. Conventional multiagent chemotherapy and radiation are usually effective, and no specific treatment protocols are available for ALPS-related lymphoma [7, 118, 119, 130]. Finally, life-threatening, early-onset lymphoproliferation may seldom represent ALPS clinical onset, requiring HSCT as the only therapeutic option. Sporadic cases have been described, often exhibiting severe GVHD even in favorable conditions [131, 132].

### Targeted treatment in ALPS-related disorders

Clinical presentation of ALPS-related disorders is often challenging and characterized by ALPS clinical signs with or without other organ-specific involvement [133]. However, autoimmune cytopenias are a hallmark of these disorders [134, 135] and are often refractory to first-line treatments (i.e., corticosteroids and IVIG) [136]. Recent evidence suggests that a rapid switchover to disease-specific therapies targeting underlying pathomechanisms is recommended [134]. Therefore, promptly achieving a genetic diagnosis is paramount in order to select a specific targeted treatment [137].

Target therapies include Janus kinase (JAK) inhibitors (e.g., ruxolitinib) and anti-IL-6 (tocilizumab) for STAT3-GoF [138], CTLA4-Ig (abatacept) for CHAI and lipopolysaccharide (LPS)-responsive and beige-like anchor protein (LRBA) deficiency [139, 140], PI3Kδ inhibitor (leniolisib) for activated PI3Kδ syndrome (APDS) [141], and others under study [134]. These treatments showed to be very effective also for other organ involvements, apart from cytopenias [138–140]. However, no targeted molecules may be available for certain disorders, and a specific molecular pathway is unknown in patients that still lack a genetic diagnosis. In these circumstances, clinical signs, immunophenotype, and laboratory parameters should guide treatment decisions [134, 135]. For instance, increased TCR αβ DNTs could be an indication to start treatment with Sirolimus, postulating a pathogenic mechanism similar to ALPS [134].

Just as in ALPS, periodic surveillance for malignancies should be carried out also in ALPS-related disorders and must be life-long, since cancer may develop at decades from disease onset (e.g., Juvenile myelomonocytic leukemia in RALD) [142]. Due to the short follow-up, it is not known whether targeted treatments may actually determine a reduction of malignant degeneration in these diseases [122]. Finally, gene editing trials are currently not available for ALPS and related syndromes, though future research might introduce this definitive treatment option, similarly to other primary immune regulatory disorders (PIRDs) [143, 144].

## Conclusions

Since its discovery 25 years ago, ALPS has become a model of monogenic autoimmunity with hundreds of disease-causing mutations in different genes being identified [8, 145]. ALPS diagnostic criteria evolved over time: the latest highlight the fact that malignant lymphoproliferation could be a warning sign of this disorder, while TCR αβ DNTs are only one of several possible disease biomarkers (Table 2) [12, 37]. Widespread use of genetic testing is revealing an increasing amount of genes hiding behind ALPS-U [36]. Moreover, an expanding number of ALPS-like syndromes are being identified, sharing clinical and immunological features with ALPS, though not completely satisfying its diagnostic criteria [16]. Concurrently, Sirolimus is becoming a first choice for treatment of ALPS [121], while targeted therapies for ALPS-related disorders are getting a foothold [134].

ALPS inheritance pattern is whatsoever complicated. According to the molecular effect of the mutation, both dominant-interference or haploinsufficiency mechanisms may develop, determining a dominant or recessive transmission, respectively [17]. The discovery of somatic *FAS* mutations as causative of ALPS furtherly complicated the plot [15]. Somatic mutations follow the same molecular mechanisms as germline ones and could present alone or in association with inherited mutations.

While heterozygous, dominant mutations in *FAS* may be sufficient to determine the disease [8], severe ALPS phenotypes often require “two hits,” which may arise in different fashions. Rarely, both of them are inherited in *FAS* or *FAS*-related genes [32, 57]. Scattered reports reveal that a “second hit” could develop in disease modifying genes that bestow pathogenic significance to heterozygous variants in *FAS*, demonstrating that a digenic inheritance model is applicable to ALPS [17, 71, 75]. Most importantly, a second somatic mutation in *FAS* — detected in TCR αβ DNTs — may often account as a “second hit,” potentially explaining the reduced penetrance of this disorder [17, 19, 56].

Due to the difficulties of performing TCR αβ DNT sorting and single-cell DNA sequencing on scant specimens [35], somatic mutations in *FAS* are probably underdiagnosed. Suspicion of somatic mutations should especially arise when facing heterozygous, non-penetrant, haploinsufficient mutations in *FAS* that inexplicably become pathogenic in an individual with healthy *FAS*-mutated family members. Moreover, somatic *FAS* mutations could potentially underlie several cases of multifactorial autoimmune diseases such as SLE, which may display clinical and immunological features that are superimposable to ALPS. Among these, an expansion of TCR αβ DNTs may occur in SLE and other autoimmune conditions: these cells could therefore represent a helpful screening tool for autoimmunity. Hence, ALPS and more common disorders may be significantly interconnected (Fig. 3), and future research may shed more light on the role of FAS signaling in both autoimmunity and cancer.

The number of discovered inborn errors of immunity (IEIs) is growing exponentially, and atypical presentations of formerly known immunodeficiencies are being progressively unmasked [145, 146]. In this context, ALPS is the prototypical example of how more genes can determine one univocal phenotype [145]. ALPS was the first example of IEI that could be also caused by somatic mosaicism. Therefore, both germline and acquired mutations can be responsible for IEIs, and research has already identified and may furtherly reveal other disorders behaving in a similar fashion [65]. ALPS could only be the tip of the iceberg.
